# Biological applications at the AQUA beamline of the EuPRAXIA@SPARC_LAB free electron laser

**DOI:** 10.1007/s00249-025-01778-4

**Published:** 2025-07-16

**Authors:** Emiliano De Santis, Tomas André, Stefania Alleva, Richard Bean, Massimo Ferrario, Augusto Marcelli, Velia Minicozzi, Emiliano Principi, Nicuşor Tîmneanu, Carl Caleman, Francesco Stellato

**Affiliations:** 1 Department of Physics, University of Rome Tor Vergata and INFN, Via della Ricerca Scientifica 1, 00133 Rome, Italy; 2 Department of Physics and Astronomy, Uppsala University, Box 516, 75120 Uppsala, Sweden; 3 European XFEL GmbH, Holzkoppel 4, 22869 Schenefeld, Germany; 4 INFN-LNF, Via Enrico Fermi, 00044 Frascati, Italy; 5 RICMASS, Rome International Center for Materials Science Superstripes, 00185 Rome, Italy; 6 ISM-CNR, Basovizza Area Science Park, Elettra Lab, 34149 Trieste, Italy; 7 Elettra-Sincrotrone Trieste S.C.p.A., Strada Statale 14 - Km 163.5, Basovizza, 34149 Trieste, Italy; 8 Center for Free-Electron Laser Science, DESY, Notkestrasse 85, 22607 Hamburg, Germany

**Keywords:** Free electron laser, Coherent diffraction imaging, Coulomb explosion imaging, Water window, Biosamples

## Abstract

The EuPRAXIA project is a European initiative aimed at developing groundbreaking, ultra-compact accelerator research infrastructures based on novel plasma acceleration concepts. The EuPRAXIA@SPARC_LAB facility, located in the Italian National Institute for Nuclear Physics-Frascati National Laboratory, will be the first operating Free Electron Laser facility of EuPRAXIA, based on an accelerator module driven by an electron bunch driver. The Free Electron Laser will produce ultra-short photon pulses in the soft X-ray region. The photons will be delivered to an endstation, called AQUA, to perform a wide range of experiments in atomic and molecular physics, chemistry, and life sciences for both academic and industrial users. Thanks to its wavelength, which falls within the so-called ‘water window’, AQUA will be particularly well-suited for coherent imaging and ion spectroscopy measurements on biological samples at room temperature in a fully hydrated environment. This unique capability opens up innovative experimental schemes for studying biological systems in states that closely resemble their physiological conditions. This paper presents numerical simulations of coherent diffraction imaging and Coulomb explosion imaging experiments, anticipating future studies at AQUA on biological samples.

## Introduction

**Fig. 1:**
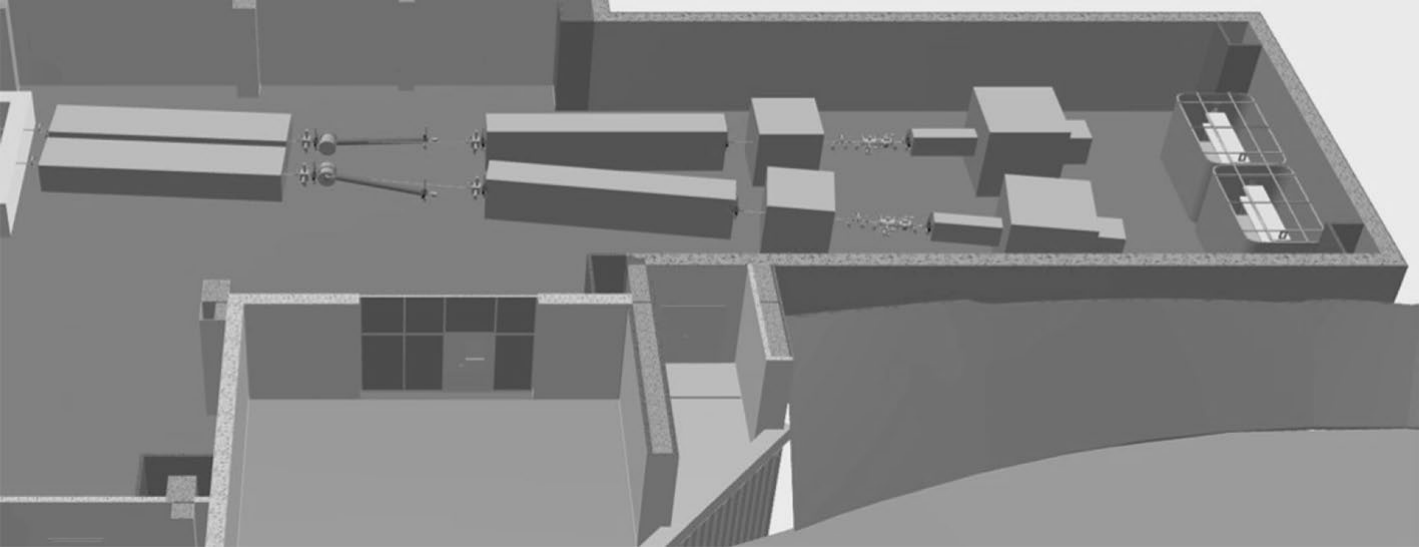
A 3D rendering of the EuPRAXIA@SPARC_LAB experimental hall, showing the two beamlines and experimental endstations

The European Plasma Research Accelerator with eXcellence In Applications (EuPRAXIA) project is aimed at developing photon sources based on innovative plasma acceleration approaches (Ferrario et al. 2018; Assmann et al. 2020). In particular, the goal is the construction of electron accelerators using laser and/or and electron-beam–driven plasma wakefield acceleration. These acceleration schemes offer a significant reduction in size—and therefore in cost—over more conventional radiofrequency-based accelerators. EuPRAXIA has received a major impulse with its inclusion in the European Strategy Forum on Research Infrastructures (ESFRI) Roadmap of 2021. EuPRAXIA foresees the construction of two plasma-based accelerators feeding a Free Electron Laser (FEL). One of this, based on an accelerator module driven by an electron bunch driver, will be built at the Italian National Institute for Nuclear Physics (INFN)-Frascati National Laboratory (LNF) (Balerna et al. 2023). This machine, called EuPRAXIA@SPARC_LAB, will provide FEL pulses with more than $10^{11}$ photons/shot, with a pulse duration of few tens of femtoseconds and a wavelength between about 4 and about 10 nm. The machine will be hosted in a 160 m long building and will deliver photons beams to two users-dedicated endstations (Fig. 1).

The primary endstation is termed AQUA, meaning water in Latin. The name comes from the fact that it will deliver photons in the water window, that is the wavelength region between the carbon and oxygen K-edge. In this region, carbon has a high cross section, while the interaction between the X-ray photons and oxygen is weak. This leads to a high contrast between the signals originated by carbon atoms, such as those contained in organic and biological molecules, and the signal coming from oxygen atoms contained in water. The AQUA endstation is currently being designed and it will allow performing a broad photon science program, spanning diverse fields as atomic and molecular physics, condensed matter and nanoscience, chemistry, including photochemistry, atmospheric and astrochemistry, and life science. Although not included in the baseline of the EuPRAXIA@SPARC_LAB project, the construction of a second accelerator branch line called ARIA (which stands for “air" in Italian), producing FEL radiation in the VUV (50 nm - 180 nm) range, is also foreseen.

Dedicated beamlines will deliver the FEL photon beam to the ARIA and AQUA experimental endstation. The AQUA beamline will characterize the FEL pulses in terms of intensity, spectral distribution, and pulse time arrival and will focus the beam down to a few micrometers with Kirkpatrick-Baez mirrors.

The AQUA endstation will be equipped with a versatile vacuum chamber to ensure experimental flexibility. Various sample delivery systems, detectors, and an external ultrafast laser for pump-probe experiments will be available, enabling a wide range of experiments, including imaging, scattering, photon spectroscopy, and ion spectroscopy.

For sample delivery, motorized holders for solid targets and injectors for liquid jets and aerosols will be installed. Liquid jet injectors are devices capable of delivering samples in a continuous stream at high velocities in a vacuum environment. These injectors are ideal for FEL experiments, where the samples are typically destroyed—or at least severely damaged—by a single photon pulse. Continuous sample delivery ensures that a fresh sample is exposed to each FEL pulse, and allows time-resolved studies of irreversible reaction and fragmentation dynamics adopting pump and probe schemes (Stellato et al. 2014; Rolles 2023; Chapman 2019; Techert et al. 2020; Garg et al. 2022; Schlathölter et al. 2016). The design of liquid injectors focuses on minimizing sample consumption while ensuring a stable and uninterrupted flow, crucial for high-resolution imaging and spectroscopy studies. The technology enables exploring molecular and atomic structures and dynamics in real time, under natural or near-natural conditions, and it is thus particularly well suited for experiments with biological specimens. AQUA will have an injector based on Gas Dynamic Virtual Nozzles (GVDNs) (DePonte et al. 2008; Nelson et al. 2016), which are able to generate ultra-thin (micron or even sub-micron), highly focused liquid jets, and are essential for delivering samples in a hydrated state, with low background and in a controlled manner (Vakili et al. 2022). The same GVDNs can also be used to feed an aerodynamic lens stacks, breaking the liquid jet into a disperse particles beam and finally focusing the resulting aerosol in the FEL beam interaction region (Bogan et al. 2010, 2010; Kirian et al. 2015). Moreover, in order to produce low-backround streams of samples, an electrospray source will also be installed. In elecrospray injection, the samples are first ionized and then sprayed into the vacuum chamber (Bielecki et al. 2019; Rafie-Zinedine et al. 2024; Roth et al. 2024). This sample aerosolization method has proven effective due to its ability to produce monodisperse, controlled, gas-phase samples. In order to provide a more thorough characterization of the injected particles, the electrospray ionization source will be coupled to a mass spectrometer, building an apparatus similar to the one developed by the European initiative MS SPIDOC (Kadek et al. 2021; Kierspel et al. 2023; Kung et al. 2025). This instrument, by filtering the particles according to their mass-over-charge ratio, will enhance sample selectivity and allow delivering low-background samples into the beam.

The endstation will be equipped with a suite of detectors to support diverse experimental needs. In particular, AQUA will host 2D photon detectors for imaging measurements, coupled with a grating to build a spectrometer for photon spectroscopy, and detectors for electrons/ions spectroscopies and ion imaging such as a Velocity Map Imaging, a Position-Sensitive Micro-Channel Plate detector (PS-MCP), a Time-of-Flight, and a Magnetic Bottle Electron spectrometer. Techniques such Coherent Diffraction Imaging (CDI), X-ray Absorption/Emission Spectroscopy (XAS/XES), and ion spectroscopy, will be implemented to perform this program (Balerna et al. 2019).

Below, we explore the opportunities that AQUA offers for studying biological systems, particularly through Coherent Diffraction Imaging (CDI) and Coulomb Explosion Imaging (CEI).

## Results and discussion

### Coherent diffraction imaging

CDI has emerged as a powerful technique in the field of X-ray science, enabling the study of non-crystalline and weakly scattering specimens at nanometer-scale resolution (Miao et al. 2011, 2015; Prosekov et al. 2021). By leveraging the high spatial coherence and extreme brightness of FEL beams, CDI measurements at FELs enable the recording of diffraction patterns from complex objects and the subsequent reconstruction of the sample’s electron density in real space without relying on crystallinity (Vartanyants et al. 2010; Mancuso et al. 2010). These measurements can be performed at room temperature, and radiation damage is naturally mitigated since, due to the very short duration and high intensity of an FEL pulse, a diffraction pattern can be recorded before significant sample damage occurs. This scheme, first demonstrated at the FLASH FEL (Beye et al. 2023) on a nanostructured non-periodic object (Chapman et al. 2006), goes beyond the name of diffraction-before-destruction (Chapman et al. 2014). CDI measurements in the diffraction-before-destruction regime will be possible at AQUA. Since AQUA will provide photons in the water window, it will allow high-resolution imaging of intact, thick hydrated samples with natural contrast (Kördel et al. 2020). Carbon-rich biological materials exhibit indeed a large difference in absorption with respect to oxygen atoms (and therefore water) in this spectral region. This characteristic makes AQUA particularly suited for high-contrast CDI of biological specimens in their native, hydrated state. Experiments at AQUA will involve injecting samples into the FEL beam within thin columns of liquid, such as liquid jets. Previous studies have demonstrated that FEL CDI measurements allow damage-free diffraction imaging of biological samples, including large viruses (Munke et al. 2016; Rose et al. 2018; Kobayashi et al. 2021), organelles (Hantke et al. 2014; Fan et al. 2022, 2024) and living cells (Van Der Schot et al. 2015; Egawa et al. 2024), at FEL facilities. Similarly, at AQUA, it will be possible to acquire damage-free diffraction patterns from large viruses, to organs and living cells, enabling reconstruction of their electron density with an expected resolution on the order of a few tens of nanometers.

**Fig. 2:**
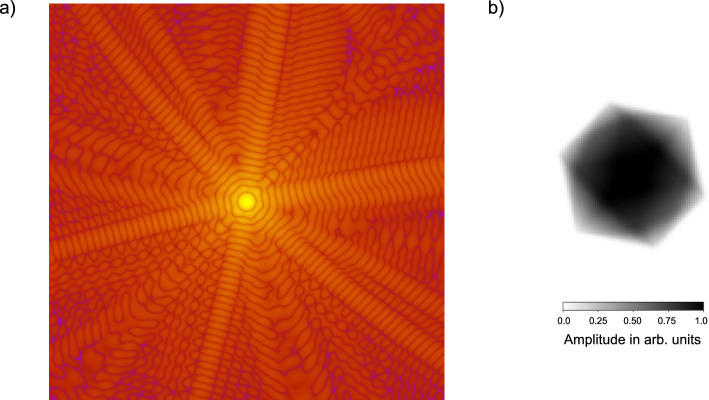
**a** Simulated diffraction pattern of an icosahedral 500 nm virus measured in the conditions described in the text. **b** Reconstructed electron density

In Fig. 2 a simulated CDI pattern of a model of the poliovirus in the AQUA experimental conditions is shown. The simulation is performed using the Condor program (Hantke et al. 2016). In particular, an FEL beam with $10^{11}$ photons/pulse with a wavelength of 4.3 nm and focused on a 5 $\mu$m spot - which is the foreseen focal spot produced by Kirkpatrick–Baez mirrors - is considered. The beam impinges on a 500 nm icosahedral virus and the diffraction pattern is acquired on a 2D detector with an array of 1024x1024 75 $\mu$m pixels located 15 cm away from the sample. With these detector and wavelength parameters, at the camera length assumed for the simulation, the electron density could (ideally) be reconstructed at a resolution of 20 nm. To illustrate the importance of performing biological imaging measurements within the water window, the fraction of photons absorbed by a 500 nm thick layer of water and a 500 nm thick layer of a virus-like material (approximated by the empirical formula $\hbox {CH}_{1.5}$ $\hbox {O}_{0.5}$ $\hbox {N}_{0.25}$ $\hbox {P}_{0.025}$Popovic 2022) has been calculated immediately above and below the carbon K-edge using the Berkeley Lab Center for X-Ray Optics online tools (Henke et al. 1993). From the experimental point of view, this corresponds to an extremely simplified schematization of a large, 500 nm diameter, virus, e.g. a mimivirus, being injected in an overall 1 $\mu$m thick water jet. Right above the carbon K-edge, at 4.3 nm, a 500 nm virus would absorb 3.3 times more than a 500 nm thick water layer, while just below, at 4.4 nm, the virus would absorb only 0.85 times as much as water. This means that, within the water window, the signal-to-background ratio increases by nearly a factor of 4.

A CDI measurement performed as described above, that is with an FEL pulse hitting - and destroying - a single viral particle, would enable the reconstruction of the 2D electron density, since a single diffraction pattern is obtained from a fixed orientation of the sample, and then the sample is destroyed. However, when multiple identical (within the achievable resolution) copies of the sample are available (this is, for example, the case of viruses (Ekeberg et al. 2024)), the combination of multiple diffraction patterns collected at various random orientations can allow the reconstruction of a 3D electron density map. It has been shown (Marklund et al. 2017; Wollter et al. 2024) that the better the sample orientation is known, the fewer patterns are required to reconstruct the 3D map. Since AQUA has a relatively low repetition rate of 100 Hz, methods for gathering information on the sample orientation in parallel with the CDI measurement could be particularly useful. In the next section, one of these methods, based on retrieving the sample orientation from the spatial distribution of the fragments created by the interaction with the beam, is described.

### Coulomb explosion imaging

One particularly interesting application of ion spectroscopy at AQUA is CEI (Unwin et al. 2023; Boll et al. 2022). By analyzing the trajectories of the ions produced by intense FEL-induced multi-ionization, CEI enables the reconstruction of three-dimensional molecular geometries and provides access to ultrafast molecular dynamics at the moment of ionization. The planned experiments will exploit this technique to capture transient structural changes in molecules undergoing chemical reactions, including isomerization, bond breaking, and complex formation. These studies will provide real-time insight into how molecular structures evolve, shedding light on reaction mechanisms and molecular dynamics. Moreover, theoretical works suggest that coupling CEI data with CDI can reveal the orientation of individual molecules and/or of larger biological complexes at the moment of X-ray irradiation, which is beneficial for improving single-particle CDI reconstructions (Östlin et al. 2018; De Santis et al. 2024; André et al. 2024, 2025).

We present results of CEI simulations for proteins under the AQUA photon pulse parameters. In CEI experiments, the samples will be delivered in the FEL beam using an aerosol injector in order to minimize the number of ions impinging on the detector, therefore optimizing the signal to noise ratio. CEI is simulated by subjecting a protein to ionization and numerically integrating the resulting atomic accelerations and velocities over time. We perform these simulations using MolDStruct (Dawod et al. 2024; André et al. 2025), an *ad hoc* modified version of GROMACS (Van Der Spoel et al. 2005), which employs a Monte Carlo approach to calculate ionization probabilities based on FEL parameters and subsequently propagates the atomic nuclei using classical molecular dynamics. Under conditions of high ionization, the protein is completely atomized; previous studies (Östlin et al. 2018; André et al. 2025; De Santis et al. 2024; André et al. 2024) have demonstrated that these explosions are highly reproducible and yield characteristic ion patterns that can be used to differentiate between the explosions of different proteins.

CEI is simulated by assuming that the AQUA beam is focused down to a micrometer spot by a multilayer off-axis parabola, such as the one successfully used at FLASH in the water window energy range (Leontowich et al. 2013). Taking into account the transmission of such a device, which is of the order of 10%, we have considered a focal spot of 1000 nm diameter and $2.3 \times 10^{10}$ photons/pulse and beam wavelength of 4 nm. These conditions result in an average ionization per atom of 0.7 *e*, which, although insufficient to fully atomize the protein, consistently induces a reproducible Coulomb explosion. We estimate that an average charge between 1 *e* and 1.5 *e* per atom is required to completely atomize a sample, depending on the specific conditions.

**Fig. 3:**
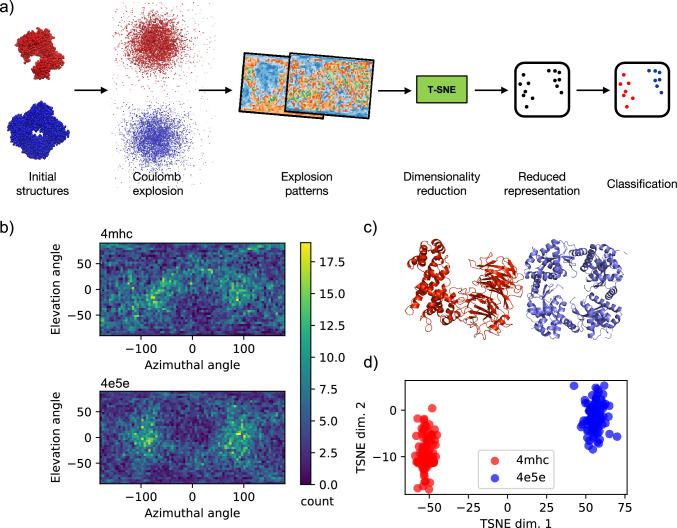
**a** Conceptual overview of the workflow: the system is first simulated under FEL pulse exposure using the MC/MD code MolDStruct (Dawod et al. 2024). From the resulting ion trajectories, explosion footprints are generated. To enable comparison, the high-dimensional footprint data is reduced in dimensionality and represented as points in a two-dimensional space. Clustering techniques are then applied in this reduced space to classify the data. **b** Explosion footprint from a 4mhc/4e5e protein simulated on a 4$\pi$-spherical detector with the angles in degrees. The colorscale represents the ion counts per bin. **c** Structure of the 4mhc (red) and 4e5e (blue) proteins. **d** Reduced space of explosion footprints of proteins 4mhc and 4e5e. Dimensionality reduction into a 2-dimensional space is performed by using the t-SNE algorithm and the units are arbitrary

An overview of the methodology used here is presented in Fig. 3a. Explosion simulations are performed for two proteins of similar mass—one from the cell nucleus (Nucleoporin, PDB ID: 4mhc Seo et al. 2013)) and one from a virus (avian influenza virus PA_N_ Apo, PDB ID: 4e5e DuBois et al. 2012). Renderings of these simulated proteins are presented in Fig. 3c. We perform 100 independent simulations per protein to assess reproducibility and provide statistical significance for pattern analysis. In these simulations, the proteins are spatially aligned and exposed to a Gaussian pulse with a 12 fs duration (full width half maximum), which corresponds to the shortest pulses produced by the EuPRAXIA@SPARC_LAB FEL. The total simulation duration is 300 fs to allow the ions to propagate freely. At the end of the simulations, when ion–ion interactions are negligible, the ions travel along linear trajectories. These trajectories are then projected onto a virtual 4$\pi$-spherical detector ($40\times 80$ elevation and azimuthal bins), mimicking an idealized PS-MCP detector, to generate a global projection visualization (see Fig. 3b). Previous analyses showed that even when approximating the idealized 4$\pi$-spherical projection with experimentally feasible planar-like detectors covering smaller solid angles, clustering accuracy remains robust, supporting the validity of this idealization (André et al. 2025). For classification, we employ t-SNE (Maaten and Hinton 2008) to reduce the dimensionality of the explosion patterns to two dimensions, plotting each event as a point in the reduced space. The resulting plot, shown in Fig. 3d, clearly distinguishes between different proteins, facilitating effective classification.

The similarity between explosion footprints from the same protein enables the inference of partial orientation, providing a practical pathway to complement traditional orientation recovery techniques in single-particle imaging. Specifically, it has been shown that pairs of explosion maps with small differences in their Euclidean distance (L2) correlate closely with small relative orientations (André et al. 2024). Current efforts aim to incorporate this partial orientation information into reconstruction algorithms for particle’s 3D diffraction intensity such as the Expand, Maximize, and Compress (EMC) (Loh and Elser 2009). A similar approach has been demonstrated by Wollter et al. (2024), who improved reconstructions by introducing orientation biases based on protein alignment with external electric fields (Sinelnikova et al. 2021).

Although the simulations presented in this work focus on single proteins, the approach could, in principle, be extended to study protein aggregates under close-to-native conditions, thus allowing the distinction between amorphous and fibrillar forms that proteins adopt under different experimental and physiological conditions (Brändén et al. 2019; De Santis et al. 2019; Popp et al. 2017; Seuring et al. 2018). CEI experiments could be performed on even larger specimes such as organelles or entire cells. However, the simulations are currently limited by computational demands that remain prohibitive due to the huge number of atoms and the complexity of charge dynamics involved. Experimentally, CEI of large biological systems has yet to be demonstrated and poses substantial challenges, particularly in resolving the dense ion clouds generated during the explosion. These challenges arise from the increased structural complexity and the difficulty of achieving the spatial and temporal resolution necessary to extract meaningful structural information from the ion distributions. However, a facility like EuPRAXIA@SPARC_LAB, with its capability to deliver ultrashort, high-intensity pulses, could serve as valuable testbed for pioneering experimental studies and driving methodological advancements in this emerging field.

## Conclusions

EuPRAXIA@SPARC_LAB will be a novel source of ultra-short, ultra-bright FEL pulses. The simulations presented in this paper indicate that CDI and CEI experiments at the AQUA beamline will be able to yield structural and dynamical information on biological specimens in their native environment.

Running CDI and CEI simultaneously turns this capability into a powerful, complementary strategy. While CDI delivers three dimensional electron density maps with spatial resolutions, CEI yields real space ion trajectory information that is sensitive to subtle conformational changes and can retain information regarding sample orientation at the moment of the interaction with the X-ray beam. Combining the two approaches therefore promises deeper insight into both shape and function within a single shot.

Time resolved measurements will push these benefits even further. Organelles—or even whole, living cells—can be probed on femtosecond time scales, alone or during infection by pathogenic agents such as viruses. The coupled CDI/CEI approach will allow intermediate states to be captured and ordered, opening a window onto the timing and mechanism of infection.

The CEI simulations reported here were performed in the gas phase. Nevertheless, a growing body of work demonstrates that proteins in vacuo can retain a near-native fold under gentle electrospray conditions (Karch et al. 2022; Marcoux and Robinson 2013; Brodmerkel et al. 2024) and subsequent molecular dynamics simulations in solvent largely recover the native structure (Brodmerkel et al. 2022, 2023). By intentionally retaining a few tightly bound water layers, the vacuum environment can be brought closer to physiological conditions. Molecular dynamics studies further predict that adding a few water layers to the protein surface reduces conformational heterogeneity (Mandl et al. 2020; Agelii et al. 2025) and sharpen the resulting CEI obtained ion maps (Östlin et al. 2018). Whether larger droplets—or a full liquid jet, as routinely used in CDI—would improve reproducibility remains to be tested.

Moreover, a key advantage of CEI proposed here is that it requires only the total ion count per solid angle and no mass spectrometric separation is necessary. This makes it realistic to extend CEI from the small molecules studied so far (few dozens of atoms) (Boll et al. 2022; Burt et al. 2017; Bhattacharyya et al. 2022; Jahnke et al. 2025; Lam et al. 2024), to much larger biomolecules and complexes.

Looking ahead, we anticipate that analysing ion trajectories from CEI will be pivotal in pushing CDI accuracy beyond what is presently feasible. With its unique pulse structure and flexible instrumentation, EuPRAXIA@SPARC_LAB offers an ideal testbed for refining and extending the combined CDI/CEI methodology, paving the way towards routine, structural biology at next generation X-ray facilities.

## Data Availability

Data supporting the results of this study are available from the corresponding authors upon reasonable request.
